# Endoscopic papillary large balloon dilation alone without sphincterotomy for the treatment of large common bile duct stones

**DOI:** 10.1186/1471-230X-11-69

**Published:** 2011-06-13

**Authors:** Hoi-Hung Chan, Kwok-Hung Lai, Chiun-Ku Lin, Wei-Lun Tsai, E-Ming Wang, Ping-I Hsu, Wen-Chi Chen, Hsien-Chung Yu, Huay-Min Wang, Feng-Woei Tsay, Cheng-chung Tsai, I-Shu Chen, Yu-chia Chen, Huei-Lung Liang, Huay-Ben Pan

**Affiliations:** 1 Division of Gastroenterology, Department of Internal Medicine, Kaohsiung Veterans General Hospital, 386 Ta-Chung 1st Road, Kaohsiung 81362, Taiwan; 2 Department of Biological Sciences, National Sun Yat-sen University, 70 Lien-Hai Road, Kaohsiung 80424, Taiwan; 3 Division of General Surgery, Department of Surgery, Kaohsiung Veterans General Hospital, 386 Ta-Chung 1st Road, Kaohsiung 81362, Taiwan; 4 Department of Radiology, Kaohsiung Veterans General Hospital, 386 Ta-Chung 1st Road, Kaohsiung 81362, Taiwan; 5 School of Medicine, National Yang-Ming University, No. 155, Sec. 2, Li-Nong Street, Pei-Tou, Taipei 112, Taiwan

## Abstract

**Background:**

Lethal pancreatitis has been reported after treatment for common bile duct stones using small endoscopic papillary balloon dilation.

**Methods:**

We retrospectively evaluated the safety and efficacy of using large balloon dilation alone without the use of sphincterotomy for the treatment of large common bile duct stones in Kaohsiung Veterans General Hospital. Success rate of stone clearance, procedure-related adverse events and incidents, frequency of mechanical lithotripsy use, and recurrent stones were recorded.

**Results:**

A total of 247 patients were reviewed in the current study. The mean age of the patients was 71.2 years. Most of them had comorbidities. Mean stone size was 16.4 mm. Among the patients, 132 (53.4%) had an intact gallbladder and 121 (49%) had a juxtapapillary diverticulum. The mean size of dilating balloon used was 13.2 mm. The mean duration of the dilating procedure was 4.7 min. There were 39 (15.8%) patients required the help of mechanical lithotripsy while retrieving the stones. The final success rate of complete retrieval of stones was 92.7%. The rate of pancreatic duct enhancement was 26.7% (66/247). There were 3 (1.2%) adverse events and 6 (2.4%) intra-procedure bleeding incidents. All patients recovered completely after conservative and endoscopic treatment respectively, and no procedure-related mortality was noted. 172 patients had a follow-up duration of more than 6 months and among these, 25 patients had recurrent common bile duct stones. It was significantly correlated to the common bile duct size (*p *= 0.036)

**Conclusions:**

Endoscopic papillary large balloon dilation alone is simple, safe, and effective in dealing with large common bile duct stones in relatively aged and debilitated patients.

## Background

Endoscopic sphincterotomy (EST) is effective in the treatment of common bile duct stones (CBDS), with complete stone removal in 85%-90% of patients[1-3]. However, EST is technically demanding and the likelihood of complications is closely related to the skill and training of the endoscopist[4].

Endoscopic papillary balloon dilation (EPBD), which is easy to perform using the wire-guided method, has become an alternative to EST for the treatment of CBDS since its first introduction by Staritz in 1982 [5]. EPBD (balloon length, 3 cm; maximum inflated outer diameter, 8 mm; and duration of inflation, 45-60 s) possesses the advantages of less bleeding, preservation of biliary sphincter function, and a reduced risk of acute cholecystitis during the follow-up period. However, for patients with difficult stones (diameter > 10 mm or number of stones > 3), mechanical lithotripsy is more frequently used after EPBD (between 10% and 50% of case), and additional sphincterotomy or repeat ERCP may also be needed[6-8]. Moreover, increased morbidity and lethal pancreatitis have been reported [9] after EPBD using a standard balloon catheter (8 mm). The authors of that study claimed that the post-EPBD pancreatitis was probably due to edematous change or trauma after the dilation procedure, resulting in the obstruction of the pancreatic duct.

Recent studies [10,11] have shown that EST followed by large balloon dilation or large balloon dilation only [12] for the removal of large or difficult stones from the CBD have good efficacy and acceptable complication rates. However, the optimal extent of EST is not known and the procedure is technically demanding when two types of sphincteroplasty are used instead of one. Theoretically, endoscopic papillary large balloon dilation (EPLBD) without EST is easier to manipulate than the combinatorial method and is also more suitable for patients with concomitant large stones and bleeding tendency. In order to evaluate the efficacy and safety of EPLBD alone in the treatment of CBDS, we conducted a retrospective study to investigate the effect of this method in patients with large CBDS in our center.

## Methods

Between September 2001 and September 2009, consecutive patients with large CBDS (> 10 mm) managed by EPLBD alone (size of dilating balloon > 10 mm, without EST) were reviewed in Kaohsiung Veterans General Hospital, Taiwan.

Local anesthesia of the pharynx was obtained using 10% xylocaine, and intramuscular injection with 40 mg hyoscine-*N*-butylbromide and 25-50 mg meperidine were administered as premedication. ERCP was performed in the standard manner using a side-view endoscope (JF-240; Olympus Optical Corporation, Tokyo, Japan). After selective cannulation of the common bile duct by the catheter, cholangiography was performed to confirm the diagnosis of CBDS. A 0.035-inch guidewire (Boston Scientific, Corp, MA, USA) was then inserted into the bile duct through the catheter. A dilating balloon (CRE balloon 5.5 cm in length, 1-1.2 cm/1.2-1.5 cm/1.5-2.0 cm in diameter; Boston Scientific, Corp, Ireland) was passed via the pre-positioned guidewire into the bile duct. Using fluoroscopic and endoscopic guidance, the balloon was inflated with sterile saline solution up to the optimal size (at least > 10 mm in diameter) and duration (usually 2-6 min) according to the patients' condition and tolerance. In order to minimize the risk of perforation, the size of the balloon should be not exceeded the size of the CBD. After removal of the balloon and guidewire, the CBDS were removed using a Dormia basket or balloon-tipped catheter with or without the aid of mechanical lithotripsy. A mechanical lithotripter (BML-4Q; Olympus Optical, Tokyo, Japan) was used to fragment the stones if they were larger than the diameter of the distal bile duct or were difficult to remove using the Dormia basket or balloon-tipped catheter. A second attempt at stone extraction was performed within 7 days if there was incomplete removal of stones in the first treatment session. All of the patients were observed in the hospital for at least 24 h after endoscopic treatment. Procedure-related adverse events and incidents were recorded according to the definitions and grading systems of the recent workshop held by the American Society of Gastrointestinal Endoscopy [13]. During the ERCP procedure, juxtapapillary diverticulum, CBD and stone sizes, stone numbers, and positive pancreatic duct enhancement were recorded. Stone removal was declared as complete if the final cholangiogram showed no residual stones. Clinical evaluation for symptoms and serum amylase was performed on the following day. Patients with complete clearance of the bile duct were assigned to regular follow-up after discharge. During each visit, a blood sample was routinely taken for liver function tests. Abdominal ultrasound was suggested every 6-12 months or at the time of abnormal liver function test or clinical symptoms suggesting the recurrence of stones. ERCP was performed if recurrent biliary symptoms, abnormal liver function, or sonographic analysis suggested recurrent CBDS. When repeat ERCP confirmed the diagnosis of recurrent CBDS, endoscopic removal of stones (with or without further balloon dilation) was performed simultaneously in the same session, or the patient was referred to the surgical department. Telephone contact was made with patients who were unable to return to the hospital.

The current study was approved by the Institutional Review Board of Kaohsiung Veterans General Hospital. The values are expressed as the mean ± SD. Categorical variables were analyzed using a χ^2^-test or Fisher's exact test and continuous variables were analyzed using Student's *t*-test. A *P *value < 0.05 was regarded as significant.

## Results

A total of 247 patients were reviewed in the current study. The patients' characteristics are shown in Table 1. The mean age of the patients was 71.2 (76% ≧ 65 years). The mean CBD size was 18.1 mm. The mean number of stones was 2.3, and the mean stone size was 16.4 mm. Of the 247 patients assessed, 132 (53.4%) had an intact gallbladder (86 had gallbladder stones) and 121 (49%) had a juxtapapillary diverticulum. Presenting symptoms included abdominal pain, 179 (72.5%); fever, 92 (37.3%); jaundice, 155 (62.8%); and pancreatitis, 18 (7.3%). The comorbidities of patients graded by the American Society for Anesthesia Classification are shown in Table 2.

**Table 1:** Patients' characteristics

Characteristics		%
Sex (M/F)	157/90	63.6/36.4
Age (mean ± SD, years)	71.2 ± 13.5 (18-98)	
Mean size of CBD (mm)	18.1 ± 5.0 (8-40)	
Mean size of CBD stones (mm)	16.4 ± 5.2 (11-39)	
Mean number of stones	2.3 ± 1.5 (1-11)	
(Single/Multiple)	88/159	35.6/64.4
Intact gallbladder	132/247	53.4
(Gallbladder stones)	86	
Juxtapapillary diverticulum	121/247	49
Symptoms		
Pain	179/247	72.5
Fever	92/247	37.3
Jaundice	155/247	62.8
Pancreatitis	18/247	7.3
WBC (× 10^3 ^cu mm^-1^)	11.0 ± 5.7	
Platelet (× 10^3 ^cu mm^-1^)	205 ± 83	
AST (IU/L)	154 ± 189	
ALT (IU/L)	160 ± 169	
Alk-P (IU/L)	298 ± 232	
r-GT (IU/L)	511 ± 445	
Total bilirubin (mg/dL)	3.6 ± 3.3	
INR (international normalized ratio)	1.06 ± 0.16	

**Table 2:** Comorbidities of patients were graded by the American Society for Anesthesia (ASA) Classification

ASA Classification	Medical description of patients	No. of patients (%)
I	No known systemic disease	115 (46.6)
II	Mild or well controlled systemic disease (s)	43 (17.4)
III	Multiple or moderately controlled systemic disease (s)	63 (25.5)
IV	Poorly controlled systemic disease (s)	26 (10.5)

Concerning the process of the EPLBD, the mean size of dilating balloon used was 13.2 mm. The duration of the dilating procedure ranged from 2 to 6 min. For 39 (15.8%) patients, mechanical lithotripsy was needed while retrieving the stones. The success rate of complete retrieval of the CBDS within the first session of treatment was 81.8% (202/247). In a further 27 patients, stones were successfully extracted in the second session of treatment (final success rate: 92.7%). Among those patients, for whom complete stone extraction was not successful (18/247), the failure was due to either stone impaction (13) or intolerance of patients (5), with seven needed placement of a plastic stent and eleven were sent for surgical treatment. The rate of pancreatic duct enhancement was 26.7% (66/247). There were 3 (1.2%) adverse events, which include 2 (0.8%) cases of mild pancreatitis and 1 (0.4%) mild cholangitis. In addition, there were six (2.4%) intra-procedure bleeding incidents. All patients recovered completely after conservative and endoscopic treatment respectively, and no procedure-related mortality was noted. A total of 172 patients had a follow-up duration of 6 months or more (mean: 30.2 ± 20.2 months), of whom, 25 patients had recurrent CBDS. The mean duration of recurrence of CBDS was 27.1 ± 17.9 months (Table 3). Table 4 showed that the recurrence of stones was not related to the age, sex, size and number of stones, use of mechanical lithotripsy and presence of juxtapapillary diverticulum (*p *> 0.05). Instead, it was significantly correlated to the CBD size (*p *= 0.036). For those who had recurrent stones, repeated ERCP with stone extraction was performed successfully in 10 patients and repeated balloon dilation was needed for accessing the stone in another 14 patients. One of the patients was sent for surgery. Among these patients, 19 presented with acute cholangitis. Moreover, 9 patients received cholecystectomy due to acute cholecystitis during the follow-up period.

**Table 3:** Results of EPLBD in the treatment of large common bile duct stones

Mean size of dilating balloon (mm)	13.2 ± 2.2 (11-20)
Mean duration of dilating procedure (min)	4.7 ± 0.7 (2-6)
Pancreatic duct visualization	66 (26.7%)
Number (rate) of mechanical lithotripsy	39/247 (15.8%)
Treatment success (first session)	202/247 (81.8%)
(second session)	27
(total)	229 (92.7%)
Adverse events	3/247 (1.2%)
Pancreatitis (mild)	2/247 (0.8%)
Cholangitis (mild)	1/247 (0.4%)
Incidents: Intra-procedure bleeding	6/247 (2.4%)
Procedure-related mortality	0
Follow-up duration ≧ 6 months	172/247
Mean duration of follow-up (months)	30.2 ± 20.2
Number of recurrent CBDS	25
Mean duration to recurrence (months)	27.1 ± 17.9

**Table 4:** Clinical parameters vs. patients with recurrent CBD stones

Clinical parameters	Recurrence (n = 25)	No recurrence (n = 222)	*P *value
Age (year)	70.0 (11.75)	71.3 (13.75)*	0.649
Sex (male:female)	14:11	143:79	0.511
Stone number	1.76 (0.93)	2.39 (1.58)	0.051
Stone size	1.63 (0.49)	1.65 (0.52)	0.896
CBD size	2.01 (0.61)	1.79 (0.49)	0.036^⋇^
Lithotripsy (+/-)	2/23	37/185	0.387
JPD (+/-)	13/12	108/114	0.834

CBD, Common bile duct.

JPD, Juxtapapillary diverticulum.

*Values expressed as the mean ± standard deviation.

^⋇^Statistically significant.

## Discussion

Since the introduction of EPBD in 1982, there has been continuous debate regarding the safety of this procedure for the treatment of CBDS. Differences in the outcomes of these procedures may be partly due to differences among the trials regarding both the inclusion criteria and the methods of preparing for and performing the procedures[6,14-20].

Apart from the aforementioned traditional EPBD method (maximum inflated outer diameter of 8 mm), large controlled radial expansion (CRE) balloon dilation (10-20 mm diameter dilating balloon), which was initially applied for dilation of the esophagus or gastric pylorus, acts as a rescue therapy after the failure of stone extraction using conventional EST plus standard basket/balloon-tipped devices due to large stone size or tapering of the distal bile duct. It has a high success rate of stone clearance, fewer complications (including pancreatitis), and minimizes the use of mechanical lithotripsy[21,22]. A subsequent randomized controlled trial [23] also demonstrated that EST plus EPLBD is a safe and effective alternative to EST alone in the treatment of CBDS. However, because sphincterotomy is arbitrary during combination therapy and the procedure is technically demanding when compared with EST or balloon dilation alone, there has been no significant decrease in lithotripsy use, even though the complication rate associated with the combined therapy is low[10].

The current study involved 247 relatively aged people (mean age: 71 years) who were suffering from considerable amounts of comorbidities. Unfortunately, most of these patients had also suffered from symptomatic large CBDS for which surgical treatment was not the first choice due to their underlying problems. These associated diseases increase the difficulty of performing ERCP procedures for patients who are also vulnerable to developing complications during conscious sedation or general anesthesia. A previous pilot study about the effect of large balloon dilation [12] reported a ballooning time (duration of dilating procedure) of 10-60 seconds, which is quite different from the 2-6 minutes in this study. However, both resulted in a low rate of pancreatitis. This further emphasizes the safety of the procedure, which is independent of the ballooning time. In addition, a recent study also demonstrated that 5-minute ballooning time improved efficacy of stone extraction and reduced the risk of pancreatitis[24].

In order to shorten the procedure time as well as minimizing the chance of complications, simplifying the procedure while maintaining the effectiveness of stone removal is warranted. Therefore, in the past few years, when we have encountered large CBD stones, we have adopted EPLBD only in our treatment scheme instead of using it in combination with EST.

We found that the success rate of bile duct clearance after EPLBD was high although repeat sessions may be needed to secure the result. Moreover, the adverse events and incidents of EPLBD are acceptable. The possible causes of the low rate of pancreatitis include, the relatively old age of the patients in the current study (mean age: 71 years),[25,26] which may be due to the progressive decline in pancreatic exocrine function associated with aging that could protect older patients from pancreatic injury. In addition, we try to selectively cannulate the CBD when performing the ERCP, and avoid cannulating or excessive injection of the pancreatic duct[27]. However, according to our experience, we sometimes had difficulty in selectively cannulate the CBD, which resulted in injecting the pancreatic duct with contrast medium accidentally. During this situation, once the head portion of pancreatic duct was filled with contrast, we would stop inject the contrast medium immediately and withdraw the catheter in order to minimize the unwanted adverse events. Moreover, large balloon dilation results in a large opening of the bile duct, which can prevent accidental cannulation of the pancreatic duct in the subsequent stone extraction and stone impaction in the common channel. Consequently, there is little need to apply mechanical lithotripsy in these cases. Although 25 patients had recurrent CBDS, they were easily handled by endoscopic treatment. Regular follow-up may be necessary, particularly for high risk patients such as those with large bile duct or poor biliary emptying [28,29].

With regard to the limitations of our current study, it suffers from the usual shortcomings associated with retrospective research, which include: a) what kind of patients should be treated by large balloon dilation, b) lack of well-defined optimal size of balloon dilation and the related ballooning time, c) complications may be underestimated, d) no standard follow-up was applied, e) relative high stone recurrence rate although a final cholangiogram was obtained to confirm the bile duct clearance for every patient, and f) significant number of patients were lost to follow-up. Further large randomized prospective studies may be needed to substantiate the true efficacy of EPLBD.

## Conclusions

Endoscopic papillary large balloon dilation alone is simple, safe, and effective in dealing with large CBDS in relatively aged and debilitated patients.

## Competing interests

The authors declare that they have no competing interests.

## Authors' contributions

HHC, KHL, CKL and PIH designed the study; HHC, KHL, and CKL analyzed the data; HHC, KHL, CKL and WLT performed the ERCP procedures; HHC, CKL, WLT, PIH, WCC, HCY, HMW, FWT were responsible for pre- and post-procedure patient care; EMW is a ERCP technician who assists the procedures; CCT, ISC and YCC are general surgeons responsible for surgical consultation; HLL and HBP were responsible for the ultrasound and CT scan interpretation; HHC and KHL were responsible for writing the manuscript and revising it critically for important intellectual content. In addition, all authors read and approved the final manuscript.

## Pre-publication history

The pre-publication history for this paper can be accessed here:

http://www.biomedcentral.com/1471-230X/11/69/prepub
